# The Sensory Landscape and Embodied Experiences in Anorexia Nervosa Treatment: An Inpatient Sensory Ethnography

**DOI:** 10.3390/jcm13237172

**Published:** 2024-11-26

**Authors:** Dimitri Chubinidze, Elisa Zesch, Amanda Sarpong, Zhuo Li, Claire Baillie, Kate Tchanturia

**Affiliations:** 1 Department of Psychological Medicine, Institute of Psychiatry, Psychology and Neuroscience (IoPPN), King’s College London, London SE5 8AB, UK; zhuo.li@kcl.ac.uk; 2 National Eating Disorder Service, South London and Maudsley NHS Foundation Trust, London SE5 8AZ, UK; elisa.zesch@slam.nhs.uk (E.Z.); amanda.sarpong@slam.nhs.uk (A.S.); claire.baillie@slam.nhs.uk (C.B.); 3 Department of Psychology, Ilia State University, 0162 Tbilisi, Georgia

**Keywords:** eating disorders, anorexia nervosa, lived experience, sensory system, ethnography

## Abstract

**Background**: Anorexia nervosa (AN) is a complex eating disorder that often requires inpatient care, where treatment experiences are influenced by both the illness and the surrounding environment. Sensory issues in AN are increasingly acknowledged for their impact on treatment engagement and outcomes. Despite this, the ways in which the sensory landscape of inpatient settings shapes patients’ lived experiences and meaning-making processes remain underexplored. **Methods**: This study employed collaborative sensory ethnography to explore how the sensory environment of an inpatient eating disorder ward shapes patients’ lived experiences. Drawing on multimodal and embodied approaches, a novel proof-of-concept method was developed, combining sensory-attuned guided reflection with AI-assisted visualization. This framework supported patients in exploring and articulating their embodied sensory experiences, linking their emotional and physical states to the ward’s sensory environment through metaphorical reasoning. **Results**: The findings reveal two central themes: a sense of entrapment within the illness and its treatment, and ambivalence toward both. The study highlights how the sensory environment and spatial layout of the ward amplify these experiences, demonstrating the tension between strict safety protocols and patients’ needs for agency and autonomy. **Conclusions**: This study illustrates the role of the sensory landscape in shaping treatment experiences and contributing to the broader lived experiences of individuals with AN. The experience of sensory cues in inpatient settings is closely intertwined with contextual and embodied meanings, often evoking complex feelings of entrapment and ambivalence toward both the illness and its treatment. These findings highlight the potential for holistic sensory and spatial adaptations in therapeutic interventions to alleviate such feelings and, consequently, improve patient engagement and well-being.

## 1. Introduction

Anorexia nervosa (AN) is an eating disorder (ED) characterized by low body weight and significant disturbances in eating behavior [1,2]. The complexity and severity of AN vary, impacting both psycho-physical and social functioning. Additionally, AN is associated with high healthcare costs and high mortality [3,4].

Given the severe health consequences, inpatient treatment for AN is often necessary, particularly for individuals who are medically unstable or unresponsive to outpatient care [5]. Treatment is typically provided by a multidisciplinary team (psychiatrists, physicians, psychologists, occupational therapists, dieticians, and nurses) and focuses on medical stabilization, nutritional rehabilitation, psychological support, and long-term recovery planning [6,7]. Treatment protocols usually include supervised meals, regular weight monitoring, and various psychological interventions [8].

Despite progress in understanding AN, research shows that optimal care management has yet to be fully achieved [5,9]. Poor treatment outcomes are often accompanied by low engagement and high drop-out rates [10,11,12]. To address these challenges, personalized treatments tailored to the individual’s needs and innovative treatment interventions are encouraged [6,12,13,14,15,16]. Understanding the lived experiences of individuals with ED is considered a crucial pathway for developing these innovations.

The research literature on the experiences of patients with AN is extensive compared to that on other EDs. Several meta-syntheses of qualitative studies highlight the shared experiences of AN patients, reflected in recurring themes [12,14,15,17,18]. However, there is relatively scarce literature on how the sensory landscape of treatment settings, particularly during inpatient admission, contribute to the overall treatment experience and meaning-making process for affected individuals.

### Sensory Perceptions and Embodiment in Anorexia Nervosa

ED research has increasingly emphasized the importance of understanding sensory issues [19,20,21,22,23,24], as sensory sensitivities can profoundly impact both the experience of the disorder and the effectiveness of treatment [25,26,27,28]. Individuals with AN often report heightened sensitivity to external stimuli such as bright lights, loud noises, and certain textures [29,30]. This hypersensitivity is linked to increased levels of ED symptoms, emotional instability, and body image issues [31,32]. Consequently, individuals with AN may feel overwhelmed and distressed in sensory-rich or chaotic environments. This distress can be further exacerbated in patients with comorbid autism or high autistic traits, which are over-represented in women with severe AN [33,34].

These sensory sensitivities necessitate further investigation into how the sensory landscape of treatment settings shape the lived experiences of individuals with AN. The term “sensory landscape” in this paper refers to the sensory characteristics and environmental stimuli within the inpatient ward, including visual, auditory, olfactory, tactile, and taste-related experiences, as well as the spatial distribution of the ward and sensory aspects of the clinical environment.

In the anthropology of eating disorders, the materialist approach to lived experiences of AN has been conceptualized as “anorexic embodiment” [35]. Informed by the embodiment paradigm [36] and other phenomenological approaches [37], this perspective moves beyond traditional clinical views, focusing on “the lived experience of being-in-the-world with anorexia, encompassing everyday practices alongside sensations, perceptions, cognitions, and concepts of identity and self” [35].

An anthropological lens examines eating disorders as co-created through the interactions between individuals and socio-cultural categories, i.e., kin, peers, treatment teams, institutional practices, societal structures, and the materialities of food, other objects, and bodies. (For further details on the socio-cultural influences and embodied experiences in eating disorders, see [38,39,40,41,42]). “Embodied experience”, as used throughout this paper, refers to the holistic felt experiences of individuals, where sensory inputs are experienced as an inseparable part of the mind, body, and environment. This concept aligns with the embodiment paradigm and the materialist approach, recognizing that physical and psychological functioning are fundamentally intertwined with the material aspects of the body and its surroundings. This study focusses on the embodied experiences during inpatient treatment to contribute to the discussion of anorexic embodiment. It aims to reveal the ways in which the sensory and material elements of the ED ward influence patients’ experiences and contribute to meaning-making during admission.

## 2. Methods

### 2.1. Setting

The study was conducted from September 2023 to May 2024 at an adult inpatient ward in a large psychiatric hospital. The hospital complex comprises several units and interconnected buildings.

### 2.2. Sensory Ethnography

A collaborative sensory ethnography (SE) was adopted. As conceptualized by Pink [43,44], SE is not merely a data collection method but a holistic approach for understanding and co-creating knowledge. This methodology combines conventional ethnographic practices, such as observation and interviews, with participatory, arts-based, and design-led methods that deeply involve participants.

The researcher’s sensory engagement and reflexivity are integral to this process. The rationale for adopting a team-based, collaborative approach to SE was twofold: to create a collaborative space with research participants and to enhance reflexivity within the research process. Collaboration with the clinical team ensures that participation is sustained, voluntary, and informed, while diverse perspectives help to enrich interpretations, especially since the research focuses on the immediate, less-representational realities of felt experiences.

### 2.3. Research Team

The research team was intentionally diversified to enhance the depth of reflexivity. It consisted of researchers and clinical team members, including a psychological anthropologist (DC), a researcher (ZL), assistant psychologists (EZ, AS), a counseling psychologist with a special interest in embodiment and eating disorders (CB), and a lead consultant psychologist (KT). While all members had professional expertise related to the field of eating disorders, DC’s lack of specific experience in this area was considered beneficial for leading the ethnography, as it was thought to enrich the study’s reflexive potential.

### 2.4. Recruitment, Data Generation, and Collection

Participants were first approached during weekly community meetings where the study and procedures were introduced. After providing consent, participants were entered into a step-by-step research process that spanned the length of their admission.

#### 2.4.1. The Sensory Wellbeing Workshop

The Sensory Wellbeing Workshop served as an immersive, collaborative groundwork for the study. It is a one-hour session designed to enhance patients’ understanding of the sensory system and its role in self-regulation (for detailed protocols see [28,45]). During the workshop, participants engaged in hands-on activities. While the workshop was not used as a primary data collection method, fieldnotes were gathered. Most importantly, it set the stage for collaborative activities, introduced participants to the researchers, and enhanced their knowledge about the sensory system through workshopping.

#### 2.4.2. Semi-Structured Interviews

Semi-structured interviews were conducted to gain an in-depth understanding of the participants’ perspectives. The interviews included open-ended questions organized into three themes: participants’ overall sensory preferences and sensitivities; how participants perceive and experience the sensory environment within the ward; and participants’ suggestions for enhancing the sensory landscape of the ward.

The interviews were carried out one-on-one and face-to-face by DC and EZ, with each interview lasting between 35 and 45 min. They were scheduled in various areas of the ward depending on space availability, such as the quiet lounge, family room, or psychological therapy room. All interviews were audio recorded and transcribed verbatim.

#### 2.4.3. Sensory Mapping and Walkthroughs

Sensory mapping and walkthroughs were conducted to pinpoint participants’ sensory experiences within the ward settings. MagicPlan app (Sensopia Inc., Montreal, QC, Canada; version 2024.5.0) was selected for its ease of use and professional capabilities in creating detailed floor plans and labeling sections of the ward.

The process of ward sketching also served as a sensory attunement exercise for the researcher, aiding in understanding the sensory landscape and spatial distribution of the ward. Using the ward plan as a base, participants were invited to map their sensory experiences by coloring different areas according to their sensory responses. They were prompted to reflect on how comfortable or distressing the sensory stimuli in each space felt, with examples provided (e.g., loud sounds, bright lights, or specific smells) to guide their color choices. Participants marked areas in red for spaces that frequently provoked strong discomfort, yellow for those that occasionally caused discomfort, and green for spaces that were consistently soothing. This sensory mapping was carried out during walkthroughs in the ward by EZ. At various points, participants paused to consider their sensory experiences in each section of the ward. EZ and AS facilitated these reflections by encouraging participants to consider both their immediate reactions and any lasting sensory impressions associated with each space. This interactive approach allowed participants to actively engage with their environment and provided a more nuanced understanding of the sensory landscape within the ward.

#### 2.4.4. Guided Sensory Reflection with AI-Assisted Visualization

Guided sensory reflection with AI-assisted visualization is a proof-of-concept method designed specifically for this study to empower patients to reflect on their treatment experience through its sensory qualities. The design of this technique was guided by discussions on the methodological dialogue between multimodality and sensory ethnography [46], with the rationale of generating data that connects sensory experiences to broader affective states of treatment. We employed a multimodal lens to allow participants to communicate meaning through multiple modes while maintaining the experiential focus of sensory ethnography, as “both are interested in meaning beyond language, in tacit and embodied knowledge and knowing” [46] (p. 97).

Participants were asked to conceptualize their time in the ward as a material object and describe its sensory characteristics. They had the option to name this object and detail its sensory qualities or follow the interviewer’s prompts regarding the object’s material attributes (e.g., shape, size, color).

For those who preferred not to participate in the guided reflection, an option to draw the object was suggested. After the reflection, the interviewer and participants agreed on the name and description of the object. Participants then explained how the object related to their ward experience. These verbal descriptions were recorded and transcribed verbatim.

Participants’ anonymized descriptions were transformed into AI requests using DALL·E (version 3; OpenAI, San Francisco, CA, USA; 2023) to visualize the objects. At least two images were generated for each description. Participants were then approached to confirm whether these visual metaphors accurately reflected their descriptions. They selected their preferred image and explained their choice. These images were incorporated into the analysis of sensory reflections to make the sensory qualities of the treatment experience more representational.

Guided sensory reflections were conducted by DC and EZ during one-on-one, face-to-face sessions lasting 20 to 25 min as participants approached discharge.

#### 2.4.5. Participant Observation

Participant observation was conducted throughout the study. DC was introduced to patients and the clinical team and attended inpatient groups, ward rounds, and the dining room, making observations for several months.

Short observational field notes were made during the research project, which were later expanded into reflexive notes. These notes captured detailed observations and reflections on the sensory experiences of both the researcher and participants.

## 3. Results

The study involved 10 adult female participants admitted to an ED inpatient ward diagnosed with AN (90%) or ARFID (10%), ranging in age from 18 to 61 years, with diverse ethnic backgrounds. Fifty percent (*n* = 5) of the participants had a comorbid condition, and 30% (*n* = 3) had either a formal diagnosis of autism or high autistic traits, as assessed by the clinical team. Additionally, 60% (*n* = 6) of the participants had an illness duration of at least three years. Not all participants were involved in every research activity, but all consented to at least three out of five activities. The flexible design of sensory ethnography accommodated the dynamic nature of the inpatient setting, ensuring inclusive participation. Refer to Table 1 for the detailed characteristics.

### 3.1. Analysis Process

The analysis was continuous throughout the study, intertwined with the research process. According to the principles of SE [44], we continuously reflected upon experiences and maintained an analytical perspective throughout. Reflexive notes generated through participant observation served as a guide during the synthesis of findings and helped maintain an analytical focus.

Data from semi-structured interviews were analyzed using reflexive thematic analysis [47,48] with initial coding done in NVivo, involving the marking of data sections and assigning of descriptors. Similar codes were then linked to form themes, transitioning the analysis from descriptive to interpretative. Themes were organized hierarchically into master and subordinate themes. For a full list of the themes, see Table 2.

Guided sensory reflections with AI-assisted visualizations produced visual images and verbal descriptions of participants’ ward experiences as material objects, capturing sensory characteristics and emotional responses. Emergent themes included the “feeling of entrapment” and the “duality of experience and emotional ambivalence”. The AI-generated images illustrated key themes, accompanied by participants’ quotes and descriptions. Notably, one image, “Fat body trapped in a small space”, was drawn by a participant, while the others were AI-generated. A summary of these findings is presented in Table 3.

**Figure 1:**
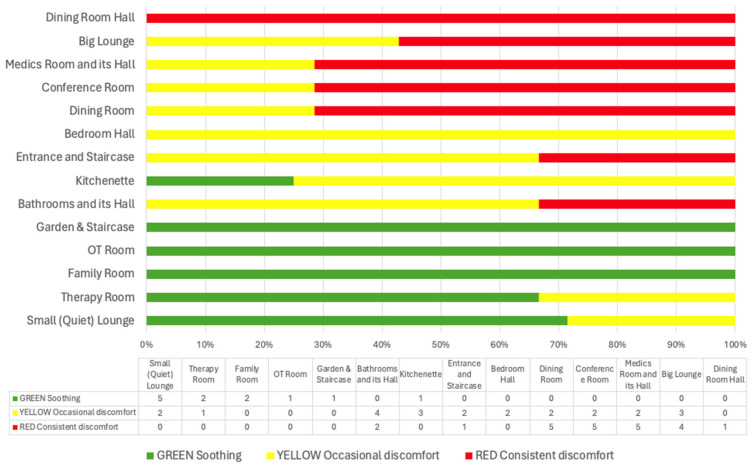
Frequency of sensory experience color codes assigned to ward spaces by participants.

**Figure 2:**
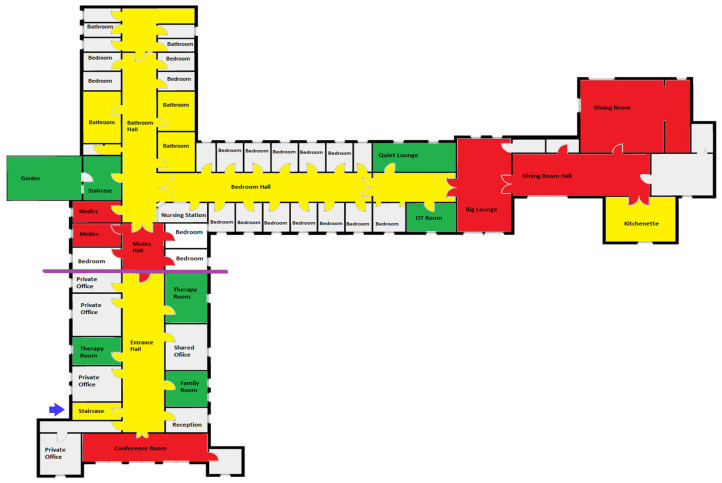
Frequency of sensory experience color codes assigned toward spaces by participants. This figure presents the composite map of the ward, illustrating the spatial distribution of sensory experiences based on the most frequently assigned color codes by participants. Each color represents different levels of sensory sensitivity: red for consistent discomfort, yellow for occasional discomfort, and green for soothing areas. Each color assignment reflects the subjective sensory experiences of participants as they engaged with different ward spaces. The purple line demarcates the separation between patient living spaces and other parts of the ward.

Sensory mapping with walkthroughs produced colored ward maps. The frequency of sensory experience color codes assigned to each ward space by the participants are presented in Figure 1. The composite map in Figure 2 reflects the consistency of responses across participants, with areas of consistent sensory discomfort (red), occasional discomfort (yellow), or soothing qualities (green) repeatedly identified, highlighting common patterns of sensory sensitivity within the ward environment.

Figure 2 presents the composite map of the ward, illustrating the spatial distribution of sensory experiences based on the most frequently assigned color codes by participants. Each color represents different levels of sensory sensitivity: red for consistent discomfort, yellow for occasional discomfort, and green for soothing areas. Each color assignment reflects the subjective sensory experiences of participants as they engaged with different ward spaces. The purple line demarcates the separation between patient living spaces and other parts of the ward.

### 3.2. Synthesis of Findings

Themes from interviews were compared with those from guided sensory reflections to identify cross-cutting themes. The higher abstraction level of themes from guided sensory reflections provided broader, metaphorical dimensions of sensory experiences and were therefore considered broader cross-cutting themes. In contrast, findings from semi-structured interviews were more descriptive and detailed, contributing to these broader themes from the guided reflections. Additionally, findings from sensory mapping were used to further evidence the integrated themes (refer to Table 2). We present the integrated findings below.

#### 3.2.1. Feeling of Entrapment—“A Fat Body Trapped in a Small Space”

Participants’ sensory experiences during inpatient admission revealed various distressing stimuli, primarily related to the ward’s safety measures, such as disturbing sounds and visuals. These unavoidable stimuli contribute to a sense of entrapment. Participants’ sensitivities to temperature, specific smells, and textures, along with their lack of control in avoiding these sensations, contribute to a restrictive environment that fosters a sense of entrapment. This feeling of entrapment is further evidenced by the sensory-attuned reflections on the overall treatment experience.

Echoes of anxiety. The auditory environment within the ward significantly impacts patients’ psychological and emotional well-being. Participants vividly described noises that heightened their anxiety and distress. Unexpected and loud sounds, such as doors slamming and staff jingling keys, acted as reminders of being in a restrictive space. One participant noted, “The sounds in the ward are very challenging for me. The noise of doors slamming and staff shouting…is extremely uncomfortable” [P2]. Another highlighted the tension created by routine practices: “Staff shouting ‘locking up’—I hate it… Ah, and those panic alarms, kind of distressing. I always put my headphones on because it has connotations, like an incident has occurred if that alarm goes on” [P1]. Another participant added, “When you hear the sound of so many keys, it is a bit like a prison” [P5].

Continuous or overlapping noises also contributed to sensory overload. P3 described the constant activity and noise around her room, saying, “It’s quite noisy in my room, so many doors are often used… It’s the multiple noises at the same time… I can’t cope with overlapping sounds. It makes me anxious and panicky… I would have a panic attack or just shut down completely” [P3]. The spatial distribution of sounds also contributes to distress, as articulated by P4: “Too much loud sound coming from different places of rooms stresses me out” [P4].

The unpredictable and diffuse nature of these sounds creates an environment where individuals feel they cannot manage their sensory input, reinforcing the sense of being in a restrictive, confining space. This auditory overload leads to heightened anxiety and a feeling of losing control, mirroring the sensation of being trapped within the ward.

Visual stressors of isolation. Participants described visual elements of the ward and expressed aversion to lighting: “Bright lights, flashing lights—I absolutely hate… especially at night-time. You can feel really alert because the lighting in there is so harsh… It’s horrible” [P4]. The desire for more subdued lighting and natural light was a recurring theme: “I would prefer it if the lights here were a bit darker… Dimmed lights… I would prefer more natural light” [P7].

Specific design features related to privacy and safety measures are also sources of visual stress. For example, the design of windows with wires is particularly distressing: “The wired windows… stress my eyes out. Like when I’m stressed, looking at those, you just want to look out the window. You can see this horrible, blurry, wired… I hate looking at them” [P4].

The combination of bright lighting and specific distressing visual features related to safety measures leads to anxiety and discomfort. The inability to escape these visual stressors parallels the sensation of being trapped within the confines of the ward.

Olfactory overwhelm. Participants described a variety of smells that trigger discomfort, headaches, and deeper psychological distress. P3 expressed her reaction to the smell of toilets, associating it with a broader emotional context: “The toilets and the smell, they’re just awful. Just makes you uncomfortable… Stale vomit. It’s not just the smell but for me, it’s you connect something with that smell. It’s a bad feeling. It just brings up this feeling of this is a really depressing place to be” [P3].

Strong smells, particularly those that are chemically based or artificially strong, were reported as sources of physical discomfort: “And just like, uh, strong smells I hate, they give me headaches… Anything that’s strong, especially like perfumes” [P4]. P1 noted, “I don’t really like really strong smells unless it’s like a smell that I really, really like and I’ve chosen… I think of like alcohol… and sanitizer and smell of gravy” [P1].

Food odors in communal dining areas can also trigger distress, as illustrated by another participant: “I also can’t stand certain smells, like in the dining room. The smell of meat is particularly disgusting to me. I hate meat, and the smell alone can disrupt everything else for me. Even plant-based burgers have a horrible smell I can’t stand” [P2]. This reflects how dietary preferences and sensitivities can extend beyond taste to olfactory experiences, influencing one’s ability to engage comfortably in communal eating settings.

During observations, it was noted that some patients expressed beliefs that food odors could contribute to their caloric intake, reflecting the extreme sensitivity and anxiety around food-related stimuli.

These sensory experiences can significantly impact psychological and emotional well-being, especially in an environment where individuals have limited control over their surroundings. When patients cannot escape or mitigate distressing odors, it could heighten feelings of discomfort, powerlessness, and entrapment, contributing to the overall sensation of being trapped within the ward.

Taste and textural discomfort. The ED ward presents challenges in addressing patients’ diverse sensory preferences, particularly regarding taste and texture.

Limitations in food texture can lead to restrictive eating behaviors, as some individuals repeatedly choose the same meals to avoid discomfort: “I have a real thing with the texture of beans because it’s hard and then it’s got the juice around it, and a lot of the things on the alternative menu had beans. So, I was like, well, if I just pick a peanut butter sandwich, then I know what it is” [P1]. This repetitive and unsatisfactory eating experience reinforces the feeling of being trapped in a cycle of discomfort.

These findings demonstrate a complex interplay between flavor preferences and texture sensitivities, impacting the dietary requirements and emotional stability. In addition to fears of weight gain and food, these sensory challenges contribute to a sense of frustration and entrapment, as patients feel unable to control or escape the distressing sensory experiences associated with inpatient treatment.

Temperature regulation challenges. This subtheme focuses on the interoceptive triggers related to internal bodily sensations such as temperature fluctuations and explores how participants experience distress related to the inability to regulate their physical environment, specifically temperature. This lack of control can exacerbate feelings of discomfort and contribute to a sense of helplessness or desire to escape. One participant detailed her struggle with the ward’s temperature, stating, “And in the showers, why is there not the function to adjust the temperature? Also being able to have radiators in every room that you can adjust temperature. I think temperature impacts my stay in the ward a lot… During isolation for being sick in a boiling hot room that was a big, big reason I meant to go home. It was just unbearable and has impacted me for not wanting to stick out…” [P3]. Another participant stated, “I guess the heat kind of stresses me out, so if it’s too hot. When you get anxious, and you get hot, you just want to be cool but it’s so hot” [P4].

The findings under this subtheme demonstrate a clear link between environmental control, specifically temperature regulation, and sense of entrapment.

Metaphors of entrapment. Guided reflections on treatment sensory experiences also reflect the sensory disturbances described above. Four multimodal sensory objects—velvet cushion of discomfort, sticky rubber ball of struggles, a fat body trapped in a small space, and isolated blue sphere—reveal intense emotional states and responses, including dislike, discomfort, aversion, frustration, and distress. These responses contribute to sensory overload and a felt experience of entrapment.

For example, P2 described the following:

“*I hate the feel and texture of velvet (expresses a shivering of dislike); it’s dry and is a very unpleasant sensation… My experience in the ward is very unpleasant. Something like being exposed to sensory stimuli you don’t like most. So touching velvet is very unpleasant for me. It’s dry. And I don’t like cushions as well. So, I relate all these unpleasant sensations to the ward*”.

Another participant, P6, drew the shape of a body confined with the title: “A fat body trapped in a small space”, conveying a profound sense of physical and emotional entrapment. Similarly, P4, with her “Sticky rubber ball of struggles”, illustrates the entrapment in a cycle: “*Kinda sticky as in like you just want something to stop but it’s just there and it just sticks… Each day it’s just there and it’s going to be tough times every day*”.

These metaphors illustrate a profound sense of physical and emotional entrapment, reflecting the meaning of participants’ inpatient treatment experiences. These metaphorical reflections offer a better understanding of how the sensory landscape of the ward contributes to the overall feeling of being trapped, providing insight into the lived sensory experiences of patients navigating their treatment.

#### 3.2.2. “Snow Leopard”—Duality of Experience and Emotional Ambivalence

The theme “duality of experience and emotional ambivalence” captures the complex emotional states associated with inpatient admission for eating disorders. This duality is reflected in the contrasting experiences of comfort and discomfort during admission and is expressed in the desire for more personalized opportunities and a sense of stability.

This duality is illustrated through the metaphor of a snow leopard, as articulated by P5. The participant uses visual and tactile sensory modes to express the dual emotional experiences linked to clinical care, the setting, and the lived experience of illness:

“*If I could put my ward experience into a material object, it would probably have to be a large soft toy, of some description, large soft toy, something like the snow leopard… Because it’s white. It would be soft and cuddly, but the animal itself is also quite a predator, so there’d always be that element of wariness. So, I would see that as my illness*”.

These predominantly tactile and visual sensory characteristics intricately encapsulate the broader ambivalent emotional experiences of safety versus vulnerability:

“*I’ve always been a huggy person, but my upbringing didn’t involve hugs. So, for me, white is pure, white is clean, and it is newness, newness of life. And although there is an element to be nearby to hug, and although deteriorating because it is a predator, and it can pounce at any time*”

[P5].

The leopard’s softness and cuddliness represent the comfort and support the participant seeks and occasionally finds in the clinical environment:

“*I think any animal looks after its young. And I think that there is an element of nurture here from the staff. That I can relate to animals when they look after their young for a period of time and then they let them go into the blue to look after themselves. And here you get the same sensation, because although I’m not their young, they’re still next to me, caring for me, teaching me the way of life. So now I can go off into the world, and explore new pathway just like, you know, a leopard or snow leopard would do that for their young*”

[P5].

Through this metaphor, we gain insight into how the participant’s embodied experience in the ward is mediated through a complex interplay of sensory modalities. The soft toy represents the participant’s perceptive engagement with her environment and condition—an engagement that is at once physical, emotional, and profoundly meaningful.

Other metaphors also highlight dual and ambivalent embodied states, each with a different focus. For example, P4’s metaphor of the “Sticky Rubber Ball of Struggles”, in addition to representing being trapped in a cycle of struggles, is also a reflection of the treatment experience as both a source of relief and a challenge: “Uh, I guess sometimes it can be helpful, for instance, because it’s cold, but sometimes it’s like the worst thing you know”.

Another participant uses the metaphor of a cracked gray square to illustrate her experience of treatment as damaging yet holding potential for usefulness: “It is rigid, and it has cracks. It’s got this problem. It should be complete, but it is damaged. But there is also the sense that there could be something inside that could be useful”. This metaphor conveys the notion of being fundamentally flawed but still holding hope for recovery or improvement.

In P1’s metaphor of a bittersweet cracker with jam, ambivalence and duality are reflected in the contradictory sensations of slimy and crunchy textures, which provoke strong aversion: “It’s like bittersweet. But then the texture is also crunchy and slimy… If I hear a crunch in a smooth thing, it’ll really upset me”. This metaphor effectively conveys the complexity of the ward experience, capturing the simultaneous existence of pleasant and unpleasant sensations that shape the participants’ emotional landscape.

Participants’ discourse reveals ambivalence toward inpatient admission, rooted in the (dis)comfort from varying levels of change and a desire for more personalized opportunities to manage and regulate the sensory landscape.

A notable example comes from a participant’s reaction to an unexpected change in the color of a commonly used item, such as cups. The participant expressed a clear preference for green or blue cups, which were familiar and therefore comforting. When presented with a purple cup, she requested it be changed back to a color she was accustomed to: “I think the colors of the cups are significant for me. We typically have green or blue cups, and one time they gave me a purple cup. I asked them to pour it into a blue or green cup because I hadn’t had a purple cup before. And they were like, ‘Why?’ And I was like, ‘I just don’t like it because I haven’t had it before’” [P1]. The importance of consistency and familiarity in personal items is also emphasized by another participant: “If I don’t like something, I simply avoid it… I don’t like changes, and I have specific sensory preferences, such as the weight of a fork or spoon, its shape, and how it feels in my hand…There’s a misunderstanding here that when I require specific types of cutleries, it’s due to my nutrition intake, but it’s really a sensory issue… I would like for people to understand that those with autism have different sensory experiences that need to be accommodated” [P2]. These instances illustrate how even minor changes in personal items can disrupt an individual’s sense of balance and comfort during inpatient admission.

The theme of duality of experience and emotional ambivalence captures the complex and often conflicting emotional states experienced by patients during inpatient admission for eating disorders. This duality is reflected in the juxtaposition of comfort and discomfort, predictability and change, and safety and vulnerability. Metaphors such as the snow leopard and the sticky rubber ball of struggles illustrate how patients navigate these contrasting experiences, seeking both care and independence while grappling with the persistent awareness of their illness. The desire for predictability and personalization reflects the patients’ need to manage the dual nature of their inpatient experience, balancing between comfort and discomfort, familiarity, and change.

#### 3.2.3. Sensory Mapping

Sensory mapping and walkthroughs of the inpatient ward revealed specific areas causing varying levels of sensory discomfort or providing soothing experiences for participants (see Figure 1).

Soothing spaces were characterized by low-intensity sensory stimulation, creating a sense of privacy. Common features included natural and dim lighting, locations in less trafficked areas of the ward, low auditory stimulation, and the ability to close doors, limiting social interactions.

Highly sensitive areas are primarily associated with eating disorder treatment processes, including eating behaviors in the dining room, and receiving feedback and defining the following week’s schedule during ward rounds held in the conference room. Clinical procedures such as blood taking, weighing, and medication distribution in the medic’s room also see high activity levels, and as one patient noted, “this area could become Clapham Junction for some”. Additionally, the big lounge is another sensitive area, serving as a multifunctional space that hosts weekly community meetings, functions as an open area between patients’ bedrooms and the dining room, and acts as a resting place post-mealtime. These areas typically involve large gatherings of people.

Mildly sensitive areas occasionally caused discomfort for some participants. Bathrooms and related halls are always locked and monitored with one-on-one observations, featuring artificial lighting, ventilation, and olfactory stimuli contributing to discomfort. Both the bottom and top entrances of staircase are locked with metal security wires, creating a sense of confinement. The halls are lit with fluorescent lighting and serve as busy paths for staff navigation, making them noisy and active.

Spatial distribution of soothing and sensitive areas. The distribution of soothing and heightened sensitivity areas in the ward reveals a fragmented sensory landscape that contributes to feelings of connection and disconnection. For example, the dining room and conference room, two highly sensitive areas, are located at opposite ends of the ward, while the medics room, another high-sensitivity area, is situated in the middle.

In between these high-sensitivity areas, there are soothing spaces such as the therapy and family rooms. However, access to these soothing spaces is limited for patients without staff and they are located outside the main patient living area. The only soothing space with unrestricted patient access is the small lounge.

This fragmented distribution of sensory environments indicates that the availability of soothing areas is both limited and unevenly spread across the ward. Consequently, patients experience a disjointed sensory journey within the ward, where moments of calm are frequently interrupted by intense sensory stimuli. This irregular availability of soothing spaces means that patients cannot consistently rely on these areas for relief, exacerbating their sense of discomfort and emotional instability.

The sensory landscape of the ward creates a cycle of connection and disconnection, leading to fragmented sensory experience, which can produce ambivalent feelings about the treatment setting. Furthermore, the high safety measures of the ward, resulting in restricted and monitored access, contribute to the feeling of entrapment.

## 4. Discussion

This study employed sensory ethnography [44] to investigate the lived experiences of treatment and how the sensory landscape of the eating-disorder inpatient unit contributes to meaning-making during admission.

The team-based sensory ethnography design established a collaborative groundwork between researchers and participants. The combination of diverse methodological techniques granted access to non-representational experiences associated with the sensory landscape, which contributes to the overall lived experience of treatment.

More specifically, guided sensory reflections with AI-assisted visualization effectively empowered patients to articulate their treatment experiences through sensory-attuned metaphorical reasoning. Participants reflected on their admission with ward-related sensory experiences to express hard-to-verbalize feelings, linking sensations to embodied affective states. It is worth noting that research generally reports difficulties in patients with eating disorders in verbalizing their emotional difficulties. Supporting patients in finding language and visual aids to express their feelings is very useful in the therapy process [49].

Furthermore, sensory mapping with walkthroughs created a path to localize sensory-induced emotional states within the ward landscape. This approach enhanced the understanding of the complex interplay between sensory stimuli and the lived experience of treatment, demonstrating the potential of combining a methodological lens of multimodality and sensory ethnography in capturing and representing embodied experiences [46].

The sensory disturbances reported in this paper are consistent with current understandings of heightened sensitivities in eating disorders, as well as with comorbid conditions [16,19,21,25,26,27,28,30,34,45]. Furthermore, both themes—“feelings of entrapment” and the “duality of experience and emotional ambivalence”—elucidated in this study strongly resonate with the lived experiences of treatment and the phenomenology of AN [12,15,18]. However, findings from this study reveal an additional sensory layer of inpatient practice contributing to these experiences.

In lived-experience research on AN, the metaphor of a trap is commonly used to describe how the illness creates a self-perpetuating cycle, where individuals lose their sense of self and feel increasingly consumed by disordered thoughts and behaviors. This concept, referred to as the “AN Trap” [50], has been further elaborated through recent interpretative, meta-ethnographic meta-syntheses of qualitative studies on severe and enduring anorexia nervosa (SE-AN) [17]. A new metaphor, a spider and its web, has been proposed to conceptualize SE-AN as a trap, contributing to the phenomenon of a disappearing self. AN is seen as an elusive defensive mechanism or tool that provides individuals with a pseudo-sense of wholeness and functionality, thus shielding them from their existential reality of isolation [15,17]. As AN dominates, it becomes self-sustaining, leading to global impoverishment where “the self becomes a shell, ghost, invisible, voiceless, or alien” [16] (p. 17).

This sense of entrapment poses significant challenges to the inpatient treatment process. The strict structure and environment can intensify feelings of entrapment from both the illness and the treatment setting. This duality can trigger behaviors of withdrawal and lack of motivation in treatment [14,51]. The findings of our study confirm this additional layer of entrapment within the sensory landscape of the ward. These cues intensify the already diminished capacity to gain control over surroundings, potentially making the treatment experience difficult. Therefore, this sensory-induced distress and feelings of entrapment may increase the treatment resistance and contribute to longer duration of illness.

Ambivalence is also a commonly reported characteristic of AN, affecting attitudes toward illness, behavioral change [52,53], and help-seeking, leading to avoidance and poor engagement in treatment [12,54]. Findings from this study suggest that the sensory landscape of the ward, with its unpredictable and broadly distributed stimuli, can create a fragmentation of soothing and unpleasant sensory experiences, further contributing to ambivalence to treatment. This sensory-induced ambivalence can, negatively impact treatment engagement.

The findings illustrate the challenges inherent within the non-negotiable context of eating disorder treatment [55]. There is an intrinsic tension between the material aspects of treatment, which aim to create an observable and safe space to prevent self-harm and monitor illness-related behavior, and the affected individuals’ lack of autonomy. Furthermore, the sensory landscape of the ward, if these embodiments are not considered, could act as maintenance factors. Fragmented, contradictory experiences reinforce ambivalence toward treatment, while safety and observability measures, along with their related sensory cues, reinforce the already-felt experience of entrapment.

The concept of anorexic embodiment, as conceptualized by Eli and Lavis [35], and the analytical lens on the material aspects of experience highlight the importance of understanding AN through an anthropological focus. This perspective can enhance the holistic adaptations of clinical practice, leading to more effective treatment approaches.

## 5. Limitations, Clinical Implications, and Future Directions

This study is primarily qualitative, and while it offers rich insights into the embodied experiences of patients with AN within inpatient settings, its findings are exploratory and may not be directly applicable to other contexts. Our findings suggest that diverse sensory cues in treatment settings are not experienced merely as isolated sensations; rather, they are mediated through contextual meanings, often embodying complex feelings of entrapment and ambivalence toward both the illness and the treatment environment.

Recognizing the sensory layers of this lived experiences can inform the development of personalized therapeutic interventions, such as sensory adaptations. Effective interventions may require a reduction in cues that reinforce illness-specific states of entrapment and ambivalence while creating adaptations that are consistent across the entire ward rather than being limited to isolated “sensory islands”. Such consistency within the sensory landscape could minimize ambivalence and contribute to a more cohesive therapeutic space.

These findings highlight the need to translate insights into sensory and spatial design adaptations within treatment settings and to evaluate their impact. Further research should assess the effectiveness of these interventions on treatment engagement and patient well-being.

## Figures and Tables

**Table 1:** Participant demographic and health characteristics and involvement in research activities.

Participant Code *	Gender	Age	Ethnicity	Diagnosis	Duration	Comorbidity	Participant Observation	Sensory Wellbeing Workshop	Semi-Structured Interview	Guided Sensory Reflection with AI Assisted Visualization	Sensory Mapping & Walkthroughs
P1	Female	19	Black/Black British—Other	AN	3 years	Autism	✔	✔	✔	✔	
P2	Female	19	White British	AN	3 years	Autism	✔	✔	✔	✔	✔
P3	Female	42	White British	AN	>3 years	EUPD; agoraphobia; chronic fatigue syndrome; PTSD; anxiety	✔	✔	✔	✔	✔
P4	Female	19	White British	AN	2 years		✔	✔	✔	✔	✔
P5	Female	61	White British	AN	>3 years		✔	✔	✔	✔	
P6	Female	20	White British	AN	2 years		✔	✔	✔	✔	✔
P7	Female	18	Mixed Race British—Other Mixed-Race	AN	<1 years		✔	✔	✔	✔	✔
P8	Female	30	White Other	AN	<1 years	Suspected EUPD and autism	✔	✔			✔
P9	Female	23	White British	AN	9 years		✔	✔	✔		✔
P10	Female	34	White British	AN	19 years	OCD; recurrent depressive disorder; EUPD	✔	✔			✔

* Abbreviations: P–participant; AN–anorexia nervosa; Autism–autism spectrum disorder; EUPD–emotionally unstable personality disorder; PTSD–post-traumatic stress disorder.

**Table 2:** Master and subordinate themes from the semi-structured interviews and cross-cutting themes from the guided sensory reflections.

Master Theme	Subordinate Theme	Cross-Cutting Themes
1. Sensory Triggers of Distress *This theme and its relevant sub-themes explore the various sensory stimuli that contribute to feelings of discomfort, anxiety, and distress. It illustrates how these sensory cues can act as constant reminders of the patients’ lack of control over their environment and reinforce the sense of being in a restrictive setting.*	1.1. Echoes of Anxiety	Feeling of Entrapment: “Fat Body Trapped in a Small Space”
	1.2. Visual Stressors of Isolation	
	1.3. Olfactory Overwhelm	
	1.4. Taste and Textural Discomfort	
	1.5. Temperature Regulation Challenges	
2. Ambivalence in Treatment: Navigating Consistency, Sensory Needs, and Personalization *This theme and its relevant sub-themes explore patients’ ambivalence toward treatment and their dual experiences of sensory stimuli. It illustrates the need for consistency in preferred sensory experiences, recognizing diverse sensory needs, and creating more opportunities to personalize the sensory landscape.*	2.1. Consistency and Predictability	“Snow Leopard”—Duality of Experience and Emotional Ambivalence
	2.2. Recognition of Sensory Needs and Personalization	

**Table 3:** Summary of findings: guided sensory reflection with AI-assisted visualization.

Sensory Object	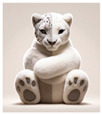 Dual Nature of Snow Leopard	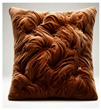 Velvet Cushion of Discomfort	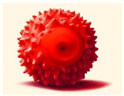 Sticky Rubber Ball of Struggles	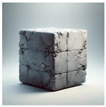 Cracked Gray Square	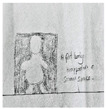 Trapped Body in a Small Space	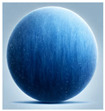 Isolated Blue Sphere	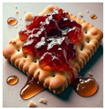 Bittersweet Cracker with Jam
Themes	Duality of experience and emotional ambivalence	Feeling of entrapment	Feeling of entrapment Duality of experience and emotional ambivalence	Feeling of entrapment Duality of experience and emotional ambivalence	Feeling of entrapment	Feeling of entrapment	Duality of experience and emotional ambivalence
Sensory Characteristics	Visual: white, large. Tactile: soft, cuddly.	Visual: brown. Tactile: dry, unpleasant. Olfactory: smell of deodorant.	Visual: round, hand-sized, red with pointy bits Tactile: rigid, sticky, cold, sweaty, clammy Auditory: loud, hollow sound Gustatory: sour orange taste	Visual: large, gray, with cracks Tactile: rigid, cold, smooth Auditory: soft, dull metallic sound	Visual: a drawing depicting a large body enclosed in a small, confined space	Visual: large, blue Tactile: hard, smooth Temperature: very cold	Visual: cracker with jam Texture: crunchy and slimy Gustatory: bittersweet, with a mix of textures
Emotional States/Responses	Feeling cared for and nurtured yet aware of potential threat. Safety vs. vulnerability. Dependence vs. independence. Comfort vs. fear.	Dislike Discomfort Aversion Frustration and distress Sensory overload Resistance	Confusion Frustration Mixed feelings of comfort and discomfort Alienation Attachment Emotional ambivalence	Sense of damage and imperfection Feeling of rigidity Curiosity and fear	Isolation	Isolation	Discomfort with mixed textures
Quotes	“It would be soft and cuddly but the animal itself is also quite a predator so there’d always be that element of weariness… “	“I hate the feel and texture of velvet; it’s dry and is a very unpleasant sensation… My experience in the ward is very unpleasant. Something like being exposed to sensory stimuli you don’t like the most.’’	“Kinda sticky as in like you just want something to stop but it’s just there and it just sticks… Each day like it’s just there are going to be tough times every day… Because it’s cold, sometimes it can be helpful, but sometimes it’s like the worst thing you know.”	“It is rigid, and it has cracks. It’s got this problem. It should be complete, but it is damaged… Just the sense that there could be something inside that could be useful”	“A fat body trapped in a small space’‘	“It’s a large circle, it’s blue, it’s cold, and it evokes feelings of being isolated because it’s distant.”	“It’s like bittersweet. But then the texture is also crunchy and slimy… If I hear a crunch in a smooth thing, it’ll really upset me.”
Participants	P5	P2	P4	P3	P6	P7	P1

## Data Availability

The data presented in this study are available on request from the corresponding author. The data are not publicly available due to ethical considerations.
